# Prevalence, heritability and genetic correlations of congenital sensorineural deafness and coat pigmentation phenotype in the English bull terrier

**DOI:** 10.1186/s12917-016-0777-6

**Published:** 2016-07-22

**Authors:** Luisa De Risio, Julia Freeman, Thomas Lewis

**Affiliations:** Neurology/Neurosurgery Unit, Centre for Small Animal Studies, Animal Health Trust, Lanwades Park, Kentford, Newmarket, Suffolk CB8 7UU UK; The Kennel Club, Clarges Street, London, W1J 8AB UK; School of Veterinary Medicine and Science, The University of Nottingham, Sutton Bonington Campus, Sutton Bonington, Leicestershire, LE12 5RD UK

**Keywords:** English bull terrier, Deafness, Pigmentation phenotypes, Heritability, Genetic correlations

## Abstract

**Background:**

Congenital sensorineural deafness (CSD) is the most common type of deafness in dogs and it occurs in numerous canine breeds including the English bull terrier. This study estimates prevalence, heritability and genetic correlations of CSD and coat pigmentation phenotypes in the English bull terrier in England.

**Results:**

Hearing status was assessed by brainstem auditory evoked response in 1060 English bull terrier puppies tested at 30–78 (mean 43.60) days of age as complete litters. Gender, coat and iris colour and parental hearing status were recorded.

The prevalence of CSD in all 1060 puppies was 10.19 % with 8.21 % unilaterally deaf and 1.98 % bilaterally deaf. The coat was predominately coloured in 49.15 % puppies and white with or without a patch in 50.85 % puppies. The majority (96.29 %) of deaf puppies had a white coat (with or without a patch); 19.29 % of the puppies with a white coat (with or without a patch) were deaf.

Heritability and genetic correlations were estimated using residual maximum likelihood. Heritability of hearing status as a trichotomous trait (bilaterally normal/unilaterally deaf/bilaterally deaf) was estimated at 0.15 to 0.16 and was significantly different to zero (*P* < 0.01). Heritability of coat pigmentation phenotype (all white/white with patches/coloured) was 0.49 (standard error 0.077). Genetic correlation of CSD with coat pigmentation phenotype was estimated at −0.36 to −0.37 (CSD associated with all white coat), but was not significantly larger than zero (*P* > 0.05). Analysis of CSD in all white and white patched puppies only estimated the heritability of CSD as 0.25 and was significantly greater than zero (*P* < 0.01), and the heritability of coat colour (all white/white with patches) as 0.20 (standard error 0.096). The genetic correlation was estimated at −0.53 to −0.54 (CSD associated with all white coat) but was just above the statistical threshold determining significant difference to zero (*P* = 0.06).

**Conclusions:**

These results indicate that CSD occurs predominantly in white English bull terriers and there is genetic variation in CSD beyond that associated with coat colour.

**Electronic supplementary material:**

The online version of this article (doi:10.1186/s12917-016-0777-6) contains supplementary material, which is available to authorized users.

## Background

Congenital sensorineural deafness (CSD) is the most common type of deafness in dogs and it has been reported in more than 90 canine breeds including the English bull terrier [1, 2]. CSD results from loss of hearing receptors in the first 3–4 weeks after birth and it is permanent [3]. Histopathological studies have revealed two main types of CSD: the cochleo-saccular and the neuroepithelial type of degeneration of the inner ear structures. The neuroepithelial type of degeneration is bilateral and characterized by degeneration of hair cells in the organ of Corti as the primary event. The stria vascularis (a modified vascular structure on the outer wall of the cochlear duct) and Reissner’s membrane are normal until the late stages of the degenerative process [2]. The vestibular structures can also be affected. This type of degeneration has been reported in the Doberman pinscher and in few other canine breeds in which no close relation between pigmentation phenotypes and deafness has been identified [2, 4, 5].

The cochleo-saccular type can occur unilaterally or bilaterally and is characterized by initial degeneration of the stria vascularis, loss of the elevated potassium concentration in the endolymph, degeneration of the hair cells within the organ of Corti, collapse of the Reissner’s membrane, degeneration and collapse of other cochlear structures including the spiral ganglion cells whose axons constitute the auditory branch of the eighth cranial nerve [2]. The vestibular structures are not affected. The cause of the stria vascularis degeneration has not yet been completely clarified, but absence of melanocytes is considered to be a main factor [2]. During embryological development, melanoblasts migrate from the neural crests to the skin, hair, eyes and stria vascularis and differentiate into melanocytes. This connection between melanocytes and cochlear function may explain the association between lack of pigmentation and CSD. Cochleo-saccular degeneration has been described in the bull terrier [6] as well as in other canine breeds displaying extensive white coat colouring [2, 7–11].

Historically, the white spotting (S) locus has been posited as playing a major role in the extent of white coat markings in dogs [12]. Four alleles have been described at the S locus: the dominant allele S producing solid colour, and three recessive alleles expressing increasing amounts of white in the coat: Irish spotting (s^i^), piebald (s^p^), and extreme white (s^w^). More recent studies have identified the microphthalmia-associated transcription factor gene (*MITF*) as being a candidate gene for, or closely linked to, the S locus [2, 13, 14], with mutations suppressing melanocytes resulting in variation in the extent of white colour of the coat as well as disrupted function in the stria vascularis leading to cochleo-saccular degeneration and deafness [2, 15]. The English bull terrier was one of the breeds used in a genome wide association study which mapped the S locus to a region within *MITF*, and identified candidate regulatory mutations in the melanocyte-specific promoter [13].

Definitive diagnosis of deafness, especially when unilateral, requires brainstem auditory evoked response (BAER) testing [2, 16, 17]. BAER is a non-invasive assessment of auditory function and it is commonly used as screening test for CSD. Auditory stimuli, in the form of clicks, are generated by computer and directed into the animal’s ear by way of specialised earphones or earplugs. The resultant response, evoked from the auditory pathway, is recorded via subdermal electrodes placed on the scalp in a specific pattern [16]. The responses are signal averaged, and the normal BAER trace consists of four or five peaks that are time-locked to the sound stimulus.

CSD can be diagnosed by BAER in dogs from 4 to 5 weeks of age [2, 17].

The prevalence of CSD (unilateral and bilateral) has been reported in various canine breeds and has ranged from 2.41 to 29.87 % [1, 18–27]. Unilateral CSD is more prevalent than bilateral CSD. The prevalence of deafness in the bull terrier has been reported as 11.00 % in one study including both puppies and adult dogs recruited in veterinary clinics and dog shows in the United States [1]. The prevalence of deafness was significantly higher in white bull terriers compared to coloured bull terriers [1]. Nevertheless, although a link between white coat colour and CSD is well established, rather than CSD fully segregating with the s^w^ allele, it merely confers an increased risk. Research groups have attempted to identify loci of genetic mutations responsible for CSD in dogs using a variety of molecular genetic techniques, however no consensus on causative gene or genes have been reached to date [28]. A reported prevalence of bilateral deafness of 2.0 % in white English Bull Terriers [1] led Karlsson et al. [13] to note that even in white dogs sufficient melanocyte migration exists to avoid bilateral CSD in most cases. Such variation in hearing status in s^w^/s^w^ homozygote animals implies underlying genetic variation, which may respond to selection. Estimation of the genetic parameters of CSD may therefore determine how viable selection against CSD could be, and reveal the extent of genetic variation beyond that directly related to coat colour.

Heritability analysis is based on the estimation of the additive genetic variation of a trait from pedigree and phenotypic (observation) records. The heritability is defined as the fraction of additive genetic variation within the phenotypic (observed) variation and so describes the extent to which the differences in breeding values (which determine the degree of resemblance between relatives) impact on variation in phenotypes, and so the reliability of phenotypic values as a guide for breeding values [29]. Thus the heritability is an important guide to the viability of selection, and whether estimated breeding values may substantially increase the accuracy of selection. The quantitative genetic methods utilised in heritability analysis may also be effective in the analysis of binary data or data with a small number of discrete ordinal categories, with the assumption of a continuous underlying distribution of liability and threshold(s) below and above which phenotypes differ [29]. CSD data, with a lower prevalence of bilateral deafness compared to unilateral deafness, appears well suited to this model. In such cases, the prevalence of each phenotypic category may be used to transform the values to more closely reflect the assumed underlying normal distribution. Where more than one trait is included in the analysis, calculation of the genetic co-variance of the two traits along with the genetic variance for each trait enables estimation of a genetic correlation, which quantifies the extent to which the genes influencing the first trait also influence the second, and so the effect selection in one trait will have on the other. Heritability estimates for CSD have been reported in Dalmatians (0.27–0.76) [21, 30–33], Jack Russell terriers (0.31) [34], Border collies (0.42) [25], and Australian Cattle dogs (0.21) [26]. Genetic correlations between CSD and white coat colour, merle coat colour, or blue irises have been investigated in Dalmatians, Border collies, and Jack Russell terriers [25, 30, 31, 33, 34].

The objectives of this study were to estimate prevalence, heritability and genetic correlations of CSD and coat pigmentation phenotypes in English bull terrier puppies undergoing BAER as part of a screening program in England.

## Methods

This study was approved by the Animal Health Trust clinical research ethics committee. The clinical database of the Neurology/Neurosurgery Service at the Animal Health Trust was searched for pure bred English bull terrier puppies that underwent BAER as part of a screening program for CSD between 10 November 1999 and 10 September 2015. The screening program was established to encourage breeders to test all surviving offsprings in a litter, including, by testing at no or reduced cost, those puppies suspected of being bilaterally deaf, based on breeder observation. Informed consent was obtained from the dog’s owners and procedures were performed in adherence to a high standard (best practice) of veterinary care. Inclusion criteria were BAER performed at 4–11 weeks of age, testing of the complete litter (except puppies which had died of natural causes), phenotypic data and Kennel Club registration information.

### Phenotypic data

The age, gender, coat colour, iris colour and hearing status based on BAER testing were recorded for each puppy. Coat pigmentation phenotype was recorded and for the purposes of this study was classified as coloured (when brindle, black, red, fawn or tricolour pigmentation predominated), all white, white with patch on head, white with patch on body, and white with patch on head and body. Iris colour was recorded as two brown eyes; one blue (partial or complete) and one brown eye; and two blue (partial or complete) eyes. The hearing status of the puppies was classified as bilaterally normal, unilaterally deaf (left), unilaterally deaf (right) or bilaterally deaf. The hearing status of both parents of each litter was stated as for the puppies, or classified as unknown. Parental hearing status was evaluated by BAER testing performed at the Animal Health Trust or at other institutions.

### BAER testing protocol

BAER recordings were performed using an electrodiagnostic machine (Sapphire 2ME 2-channel system, Medelec, or Synergy N-EP 5-channel system, Medelec) without sedation or anaesthesia. The amplifier settings for the Sapphire 2ME were 20 μV per division, and for the Synergy were 10 μV per division, with a sweep duration of 10 ms for both machines. Low frequency cut off was 100 Hz, with a high frequency cut off of 2 kHz for the Sapphire 2ME and 3 kHz for the Synergy N-EP. Stainless steel needle electrodes (12 mm long, 0.3 mm diameter) were inserted under the skin at three sites on the head. The ground electrode was situated over the occipital protruberance, the reference electrode at the vertex and the recording electrode just rostral to the tragus of the tested ear. Rarefaction or alternating clicks, 0.1 ms in duration were delivered at 80 dB nHL, via an unshielded audiometric headphone (model TDH49P, Medelec) held against the opening of the external auditory meatus. Recordings were acquired at a click rate of 20–30/s. No fewer than 512 responses were signal averaged to eliminate artefact. If no response was elicited at 80 dB, then the test was repeated at 100 dB. To exclude the contribution from a normal-hearing contralateral ear, white noise was presented, at 20 dB less than the stimulus level, into the non-stimulated ear. This prevented the stimulus from the examinad ear being registered by the opposite ear. Both ears were tested in turn, right ear first then left ear.

### Pedigree

Using the unique Kennel Club registration number, phenotypic data records were linked to the Kennel Club English bull terrier pedigree database. The pedigree database contains 90,342 registration details of all English bull terriers and includes: unique alpha-numeric registration number, registered name, gender, date of birth, coat colour, sire registration name and number and dam registration name and number. Tested puppies were linked to the Kennel Club database via parental registration numbers, since not all puppies in a litter were always registered with the Kennel Club prior to BAER testing. The entire Kennel Club electronically held pedigree was used in the genetic analysis, with English bull terrier puppies born in 1999 and 2015 (the year of birth boundaries of puppies undergoing BAER screening in this study) having an average of 16.06 and 21.53 generations of pedigree respectively.

### Estimation of genetic parameters

Mixed linear models using ASREML [35] were fitted to the hearing status data (0 = bilaterally normal, 1 = unilaterally deaf, 2 = bilaterally deaf) for *n* = 1060 puppies to estimate variance components. The general form of the linear model was as follows:

$$ \mathrm{Y} = \mathrm{X}\mathrm{b} + \mathrm{Z}\mathrm{a} + \mathrm{e} $$

where Y is the vector of observations, X and Z are known incidence matrices, b is the vector of fixed effects, a is the vector of random additive genetic effects with the distribution assumed to be multivariate normal, with parameters (0, σ^2^_a,_**A**) and e is the vector of residuals distributed with parameters (0, σ^2^_e,_ I). I is an identity matrix of the appropriate size, A is the additive genetic relationship matrix and σ^2^ denotes the variance of each of the respective random effects. Preliminary models indicated that inclusion of litter and dam as random effects in the mixed model analysis of hearing status were not significant (*P* > 0.05, likelihood ratio test). The fixed effects included in the model were gender, year of test and coat pigmentation phenotype. Only 15 animals were contained within the coat colour categories ‘white with body patch’ and ‘white with body and head patch’, so these were combined with the category ‘white with head patch’ to give three coat pigmentation phenotype categories to be used in the analysis: completely white (0, *n* = 188), white with patches (1, *n* = 351), and coloured (2, *n* = 521). Age (in days) at test and inbreeding coefficient were included as covariates. Iris colour was not included in the analyses as only two of 1060 puppies had unilateral blue irises, the remainder having bilaterally brown irises.

### Liability transformation

The hearing status data were transformed to reflect the presumed underlying normally distributed genetic liability of CSD based on proportion of hearing, uni- and bi-laterally deaf categories (0 [bilaterally normal hearing status] = −0.195; 1 [unilaterally deaf] = 1.589; 2 [bilaterally deaf] = 2.421), [29], and analyses repeated using this as the dependent variable.

### Multivariate analysis

Multivariate analysis was performed with the dependent variables hearing status (separately on the observed and liability scale) and coat pigmentation phenotype (0 = completely white, 1 = white with patches, 2 = coloured) included in an animal model, with fixed effects as previously described. The variances (σ^2^) were replaced with the 2x2 variance/covariance matrix for both traits and the direct product operator. The heritability for each trait and genetic and residual correlations between each pair of traits was estimated.

The phenotypic variance, denoted as σ^2^_*p*_, comprises the sum of the additive genetic variance and residual variance (σ^2^_*a*_ + σ^2^_*e*_). The heritability (h^2^) is calculated as the proportion of the phenotypic variance explained by the additive genetic variance (σ^2^_*a*_ / σ^2^_*p*_). In multivariate analysis the additive genetic variance of each trait and the covariances between each pair of traits are used to calculate the genetic correlation:

$$ {r}_{A\left(a,b\right)}=\frac{\sigma_{A\left(a,b\right)}}{\sqrt{\sigma_{Aa}^2}\cdot {\sigma}_{Ab}^2} $$

where r_A_ is the additive genetic correlation, *a* and *b* denote the two traits in question, σ^2^_A_ denotes the additive genetic variance of traits and σ_*A(a,b)*_ is the additive genetic covariance of trait *a* with *b*.

### Analysis in dogs with completely or predominantly white coat

CSD has been shown to be associated with extensive white coat colour [28], associated with the s^w^/s^w^ genotype known to be prevalent in the English bull terrier, yet variation in hearing status beyond the genotype at the S locus is evident [13]. Therefore, the analyses described above were repeated excluding dogs with coloured coat (*n* = 521), in an attempt to determine the extent of genetic variation in hearing status only in dogs with completely or predominantly white coat (e.g. all white or white with one or two small coloured patches) (*n* = 539), and so presumed to possess the s^w^ allele. For these analyses liability transformed scores were 0 [bilaterally normal hearing status] = −0.335; 1 [unilaterally deaf] = 1.252; 2 [bilaterally deaf] = 2.208, to reflect the different categorical proportions of CSD in the reduced data.

## Results

### Prevalence

**Fig. 1:**
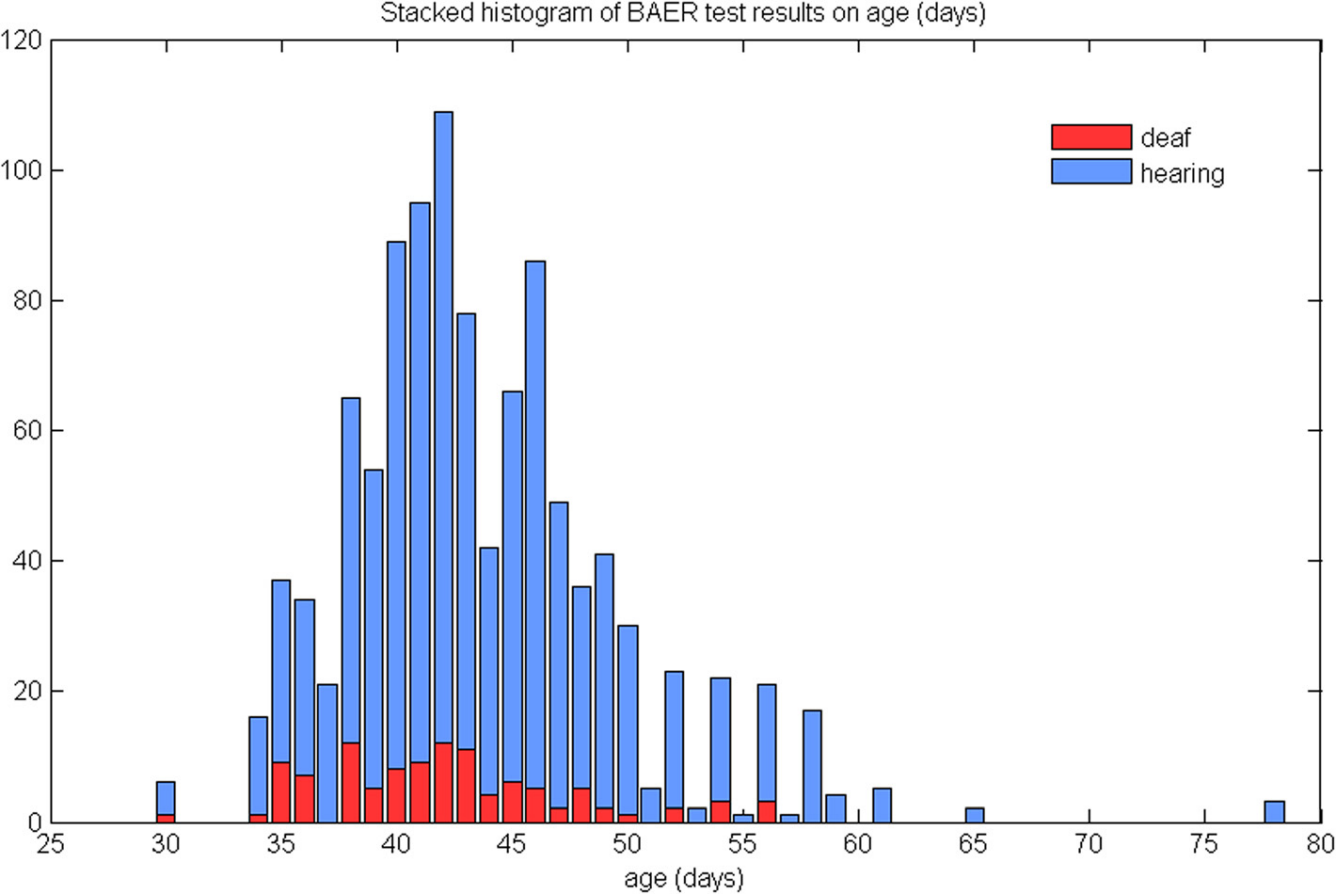
Stacked histogram showing age (in days) at time of BAER testing and hearing status (normal hearing status versus uni- or bi-lateral deafness) in 1060 English bull terrier puppies

Inclusion criteria were met for 1060 pure bred English Bull terrier puppies from 209 unique litters (Additional file 1). There were 100 unique sires and 148 unique dams. The litters were from different regions of England. Age ranged from 30 to 78 days (mean 43.60, SD 5.85 days). Hearing status on age is shown in Fig. 1. Year of BAER testing ranged from 1999 to 2015 (*n* = 6, 6, 9, 24, 34, 48, 30, 56, 103, 118, 99, 86, 131, 98, 113, 62, 37 puppies). Hearing status was bilaterally normal in 952 (89.81 %) puppies, 87 (8.21 %) were unilaterally deaf and 21 (1.98 %) were bilaterally deaf. Of the 87 puppies with unilateral CSD, 33 (37.93 %) were deaf in the left ear and 54 (62.07 %) were deaf in the right ear. There were 547 (51.60 %) males and 513 (48.40 %) females. Puppy hearing status stratified by coat colour, gender and hearing status of sires and dams is summarised in Table 1. Irises were brown in all but two puppies with one blue iris each (left blue *n* = 1; right blue *n* = 1). These two puppies with blue irises were 38 and 40 days old at time of BAER, respectively; one was unilaterally deaf and one had normal hearing status. Coat colour in the two puppies with blue irises was all white and white with a patch on the head, respectively. None of the sires and dams with known hearing status was unilaterally or bilaterally deaf.

**Table 1:** Cross tabulation of hearing status in 1060 English Bull terrier puppies by coat colour, iris colour, gender and parental hearing status

		Puppy hearing status			Total
		Normal	Unilateral deafness	Bilateral deafness	
Coat colour	Coloured	517 (99.23 %)	3 (0.58 %)	1 (0.19 %)	521
	White	149 (79.26 %)	29 (15.43 %)	10 (5.32 %)	188
	White with head patch	271 (80.65 %)	55 (16.37 %)	10 (2.98 %)	336
	White with body patch	5 (100.00 %)	0 (0.00 %)	0 (0.00 %)	5
	White with head and body patch	10 (100.00 %)	0 (0.00 %)	0 (0.00 %)	10
Gender	Male	494 (90.31 %)	43 (7.86 %)	10 (1.83 %)	547
	Female	458 (89.28 %)	44 (8.58 %)	11 (2.14 %)	513
Parental hearing status	Normal in both parents	436 (91.60 %)	34 (7.14 %)	6 (1.26 %)	476
	Normal in sire and unknown in dam	227 (89.37 %)	20 (7.87 %)	7 (2.76 %)	254
	Normal in dam and unknown in sire	108 (94.74 %)	4 (3.51 %)	2 (1.75 %)	114
	Unknown in both parents	181 (83.80 %)	29 (13.43 %)	6 (2.78 %)	216

### Results of genetic analysis in all dogs

There was no effect of year of BAER testing, sex or inbreeding coefficient on hearing status (ANOVA tables included in Additional file 2). There was no significant effect of age on hearing status, and the estimated effect was small, equivalent to +0.20 and +0.32 from 30 to 78 days (the limits of the age range in this study) on the observed and liability scale respectively. Coat pigmentation phenotype (completely white, white with patches, or coloured) was associated with hearing status in univariate analyses, with coloured dogs significantly less likely to be deaf compared to all white dogs (−0.268, standard error 0.032 on the observed scale; −0.429, standard error 0.050 on the liability scale). Patched white coat had a similar and not significantly different effect on hearing status to all white coat (−0.043, standard error 0.033 on the observed scale; −0.054, standard error 0.0519 on the liability scale).

The estimate of heritability of hearing status was 0.151 (0.059) on the observed scale and 0.159 (0.061) on the liability scale. Estimates were significantly larger than zero (*P* < 0.01, likelihood ratio test). Univariate analysis of coat pigmentation phenotype (0 = completely white, 1 = white with patches, 2 = coloured) estimated the heritability at 0.488 (0.077). When hearing status and coat pigmentation phenotype were analysed in a bivariate model, the estimates of heritability were of similar magnitude to those estimated using univariate models (0.141 and 0.489 with hearing status on the observed scale; 0.144 and 0.487 with hearing status on the liability scale). The genetic correlation between hearing status and coat pigmentation phenotype was −0.372 (0.185) with hearing status on the observed scale and −0.358 (0.186) on the liability scale (i.e. CSD associated with completely white coat). However on neither scale was the genetic correlation significantly larger than zero (*P* > 0.05, likelihood ratio test). The residual correlation between hearing status and coat pigmentation phenotype was similar, although slightly smaller in magnitude, and was significantly larger than zero (*P* < 0.01; −0.276, standard error 0.053 on the observed scale; −0.292, standard error 0.053 on the liability scale).

### Results of genetic analysis in dogs with completely or predominantly white coat

Univariate analysis of hearing status (0 = bilaterally normal, 1 = unilaterally deaf, 2 = bilaterally deaf) using data from all white and white patched dogs only (*n* = 539) determined heritability estimates of 0.245 (standard error 0.108) and 0.254 (standard error 0.102) on the observed and liability scale respectively. Both estimates were significantly greater than zero (*P* < 0.01, likelihood ratio test). Univariate analysis of coat pigmentation phenotype (0 = completely white, 1 = white with patches) estimated the heritability at 0.196 (standard error 0.096), implying additive genetic variation in the presence and absence of patches of pigment in white dogs. Bivariate analysis of hearing status and coat pigmentation phenotype (0 = completely white, 1 = white with patches) yielded slightly higher heritability estimates than from the univariate analysis (0.306 and 0.238 respectively with hearing status on the observed scale; and 0.320 and 0.239 respectively with hearing status on the liability scale). However, estimates were within one standard error of those estimated in univariate analyses. The genetic correlation between hearing status and coat pigmentation phenotype (0 = completely white, 1 = white with patches) was −0.544 (standard error 0.240) with hearing status on the observed scale and −0.528 (standard error 0.239) on the liability scale (i.e. CSD associated with completely white coat). In both cases the genetic correlation was very close to the threshold indicating estimates were significantly larger than zero (*P* = 0.06, likelihood ratio test). Residual correlations were smaller in magnitude and not significantly larger than zero; 0.087 (standard error 0.081) on the observed scale, and 0.093 (standard error 0.083) on the liability scale.

## Discussion

The overall prevalence of CSD in the 1060 English bull terrier puppies included in this study is 10.19 %, which is similar to the prevalence of deafness (11.00 %) reported in a previous study including 665 English bull terriers [1]. Also the prevalence of unilateral and bilateral deafness is comparable between this study (8.21 % and 1.98 %, respectively) and the study by Strain (9.90 % and 1.10 %, respectively) [1], although the proportion of dogs with unilateral deafness is 1.69 % lower in our study. In both studies the majority of deaf dogs had a white coat (with or without a patch), being 96.29 % and 94.52 % of the total number of deaf dogs in this study and in the study by Strain [1], respectively. The proportion of dogs with blues irises is very low (1.88 % and 1.50 % respectively) in both studies [1] and therefore association with CSD could not be assessed. Blue or partly blue irises are an undesirable phenotype in the English bull terrier and therefore selective breeding has minimised its occurrence. No significant gender difference was seen in CSD prevalence in either study [1]. Comparison between our study and the study by Strain [1] should be performed considering the differences in population sampling and inclusion criteria. Our study included only complete litters of puppies undergoing BAER as part of a screening program whereas the study by Strain [1] included dogs of any age recruited at veterinary clinics and dog shows, including single dogs with suspected hearing deficits. This latter difference is the most likely explanation for the higher prevalence of bilateral deafness in the study by Strain [1] compared to ours.

Similarly to previous studies in other canine breeds with CSD [20, 23], prevalence of CSD (unilateral or bilateral) is higher in English bull terriers with unknown hearing status in both parents (16.20 %) compared to English bull terriers with normal hearing parents (8.40 %). However CSD still occurred in the offspring of BAER-tested normal parents, therefore additional breeding strategies are required to reduce the prevalence of CSD. It has recently been suggested that exclusive breeding of litters in which both parents and all four grandparents are BAER-tested normal is expected to reduce CSD prevalence in pups to the greatest extent over the long term [36].

The prevalence of unilateral and bilateral CSD in coloured English bull terrier puppies was 27 and 19 times lower respectively than that in the other two predominantly white combined categories (‘white’ and ‘patched’). The significance of the coloured coat pigmentation phenotype as a fixed effect in the univariate analysis of hearing status, with coloured English bull terriers less likely to be deaf than either all white or white patched English bull terriers, is in line with the dual role of melanocytes in cochlear function and extent of coat pigmentation. Therefore, breeding only coloured English bull terriers would help to minimise the breed-wide occurrence of CSD. However, in this study, 80.71 % of the puppies with a white coat (with or without a patch) also had bilaterally normal hearing status, demonstrating that a white coat does not definitively imply occurrence of CSD in English bull terriers, and implying some other influence (genetic or otherwise) in the manifestation of CSD.

The categories of presence and extent of coat pigmentation utilised in this analysis reflect allelic variations at the white spotting locus (*MITF*), where the dominant allele S produces solid colour, and the recessive allele s^w^ results in an extreme white coat phenotype, with occasional small areas of pigmentation (observed and categorised as ‘patches’ in this study). The extreme white allele, s^w^ is known to produce the extreme white coat in the English bull terrier [2, 13]. The prevalence of CSD in completely white and white patched puppies was similar (15.43 % vs 15.67 % unilateral CSD, and 5.32 % vs 2.85 % bilateral CSD) and the fixed effects on CSD of the ‘white’ and ‘patched’ coat pigmentation categories appeared equivalent. Thus, there is evidence that the ‘white’ and ‘patched’ pigmentation phenotypes may be considered to be a single category of ‘extreme white’, particularly given the known variation in pigment phenotypes of s^w^/s^w^ dogs outlined above. Therefore, the genotype s^w^/s^w^ was presumed in puppies with the ‘white’ and ‘patched’ phenotypes and the analysis of this cohort alone was undertaken to determine any additive genetic variation in predisposition to CSD beyond that conferred by the s^w^ allele.

The estimate of the heritability of CSD as a trichotomous trait reported in our study is 0.15–0.16 over all coat pigmentation phenotypes and 0.25 in dogs with completely white and patched white coats. These results suggest that additive genetic variation in dogs included in this study accounts for a significant proportion of the phenotypic variation in CSD observed and implies that genetic selection away from CSD would be successful, even in English Bull Terriers with the extreme white coat phenotype. Furthermore, the magnitude of the heritability estimated in this study implies substantial improvement in the accuracy of selection against CSD may be achieved via the use of estimated breeding values, resulting in a concomitant increase in the response observed. Previous studies of CSD have reported heritability estimates ranging from 0.21 in Australian Cattle dogs [26] to 0.76 in Dalmatians [31], although direct comparison of heritability of CSD in different populations of breeds is complicated by variations in population size, sampling methods, breeding standards and prevalence of deafness within each population. Furthermore, additive genetic variance, and so the heritability, are parameters unique to specific populations since they are dependent on gene frequencies therein [29].

The genetic correlation between hearing status and coat pigmentation phenotype determined using all dogs (*n* = 1060) in this study was sizable (−0.35 to −0.37), implying a genetic association between white coat colour and CSD which reflects the discrepancy in prevalence of CSD in coloured and predominantly white dogs (0.77 % vs 19.29 %) and is likely to be largely due to the effect of the S allele in coloured dogs. However, the genetic correlation was not significantly different to zero, which may be due to the large effect of a single gene. When the analyses were repeated using only dogs with the presumed genotype s^w^/s^w^ (‘white’ and ‘patched’), this study determined that there was still significant additive genetic variation and so a sizable estimate of heritability of CSD in completely or predominantly white dogs. This means there is variation in genetic predisposition to CSD in the English Bull Terrier beyond that conferred by the alleles at the white spotting locus in *MITF*. The presence of pigmentation in predominantly white dogs (i.e. patching) was also found to be moderately heritable, implying the existence of genetic modifiers overlaying the major effect of the extreme white (s^w^) allele. The genetic correlation between hearing status and presence/ absence of a patch in white dogs was moderate and implies a genetic association between CSD and completely white coat (compared to some small patches of pigmentation), although falling just short of the threshold determining significance (from zero), most probably due to a small sample size (by quantitative genetic standards).

## Conclusions

The overall prevalence of CSD in the 1060 English bull terrier included in this study was 10.19 %, similar to that reported previously in this breed. This study has successfully demonstrated the existence of underlying additive genetic variation in the risk of CSD in predominantly white English Bull Terriers (presumed to have the genotype s^w^/s^w^), and this can be the focus of selection to improve canine welfare. The estimated genetic correlations imply probable genetic commonality between deafness and white coat, even among white patched dogs, although estimates were not significantly different to zero (possibly due to a relatively small sample size).

## Abbreviations

BAER, brainstem auditory evoked response; CSD, congenital sensorineural deafness; *MITF*, microphthalmia-associated transcription factor gene

## Additional files

Additional file 1: Phenotypic data of 1060 English bull terrier puppies included in the study. Phenotypic data file, including animal identification numbers, effects and phenotypic data; fields are: datid – data point identity number; pid – pedigree identity number; ps – sire pedigree identity number; pd – dam pedigree identity number; litter – litter number; f – inbreeding coefficient; sex – 0 = male, 1 = female; age – age in days at BAER test; yot – year of BAER test; patch – location of patches; 0 = solid coloured, 1 = all white, 2 = white with patch on head, 3 = white with patch on body, 4 = white with patches on head and body. mel – pigmentation categorisation 1; 0 = all white, 1 = patch on head only, 2 = patch on body, 3 = solid coloured. mel1 – pigmentation categorisation 2; 0 = all white, 1 = non-white (including patched); mel2 – pigmentation categorisation 3; 0 = all white, 1 = patched, 2 = solid coloured; deaf1 – hearing status categorisation 1; 0 = bilaterally hearing, 1 = uni- or bilaterally deaf; deaf2 – hearing status categorisation 2; 0 = bilaterally hearing, 1 = unilaterally deaf, 2 = bilaterally deaf. (TXT 59 kb) Additional file 2: Anova tables showing significance of effects in the mixed model. Effects in mixed model, degrees of freedom and F values. (DOCX 14 kb)
