# Supplementary material for: Prevalence, heritability and genetic correlations of congenital sensorineural deafness and coat pigmentation phenotype in the English bull terrier
Source: BMC Vet Res. 2016 Jul 22;12:146. doi: 10.1186/s12917-016-0777-6 (PMC4957289; doi:10.1186/s12917-016-0777-6)
Supplement: Additional file 2: — Anova tables showing significance of effects in the mixed model. Effects in mixed model, degrees of freedom and F values. (DOCX 14 kb) [file 12917_2016_777_MOESM2_ESM.docx]

Additional file 2. Anova tables showing significance of effects in the mixed model.

| Source of Variation | NumDF | F-inc |
| --- | --- | --- |
| mu | 1 | 5.94 |
| sex | 1 | 0.38 |
| year of BAER test | 16 | 1.05 |
| inbreeding coefficient | 1 | 0.04 |
| coat colour | 2 | 51.48 |
| age (days) | 1 | 2.96 (Observed scale) |

| Source of Variation | NumDF | F-inc |
| --- | --- | --- |
| mu | 1 | 0.33 |
| sex | 1 | 0.33 |
| year of BAER test | 16 | 1.19 |
| inbreeding coefficient | 1 | 0.05 |
| coat colour | 2 | 56.75 |
| age (days) | 1 | 3.13 (Liability scale) |
